# Circadian rhythm in restless legs syndrome

**DOI:** 10.3389/fneur.2023.1105463

**Published:** 2023-02-23

**Authors:** Mingyang Tang, Qingqing Sun, Yanan Zhang, Huimin Li, Dong Wang, Ying Wang, Zan Wang

**Affiliations:** Sleep Center, Department of Neurology, The First Hospital of Jilin University, Changchun, Jilin, China

**Keywords:** restless legs syndrome (RLS), circadian rhythm, brain iron, dopamine, hormones

## Abstract

Restless legs syndrome (RLS) is a sensorimotor disorder with a obvious circadian rhythm, as its symptoms often occur or worsen only in the evening or at night. The mechanisms behind the rhythms of RLS have not yet been fully elucidated. This review explores possible causes for the circadian fluctuations of the symptomatology, including the levels of iron, dopamine, melatonin, melanocortin, and thyroid-stimulating hormone in the brain, as well as conditions such as peripheral hypoxia and microvascular function disorders. The metabolic disturbances of the substances above can create a pathological imbalance, which is further aggravated by physiological fluctuations of circadian rhythms, and results in the worsening of RLS symptoms at night. The review concludes with the suggestions for RLS treatment and research directions in the future.

## 1. Introduction

Restless legs syndrome (RLS) is a common sensorimotor disorder characterized by strong and persistent urges to move. It is often accompanied by uncomfortable and varied limb sensations that may be described as creeping, itching, or pulling; these occur or worsen in the evening/night when at rest and may resolve after movement. RLS is often accompanied by periodic limb movements at night, which can result in disturbed sleep architecture and frequent awakenings (1). RLS may cause severe difficulty in falling asleep, poor sleep quality, impaired daytime function, increased incidence of cardiovascular or cerebrovascular disease (2, 3), and diminished quality of life.

The mechanisms underlying RLS have not yet been fully elucidated. Possible mechanisms include iron deficiency, altered dopaminergic function (4), and specifically increased excitability of the glutamate system in the brain; which can cause central nervous system sensitization and hyperarousal, resulting in sensory disturbances and frequent awakenings (5–7). Adenosine has also been implicated: a severe iron deficiency may downregulate the adenosine A1 receptor (A1R) and upregulate the adenosine A2A receptor (A2AR), resulting in a hypoadenosinergic state and an increase of A2ARs that do not form heterodimers with A1Rs, while a milder iron deficiency may only affect A1R. This increases the sensitivity of cortico-striatal glutamatergic terminals and promotes a hyperglutaminergic state (8, 9).

The opioid system is also involved in RLS pathogenesis. Walters et al. found statistically significant reductions in β-endorphin and Met-enkephalin levels of patients with RLS (10). The effectiveness of opioid receptor agonists demonstrate the therapeutic impact of these receptors, and receptor knockdown can cause iron deficiency anemia, dopaminergic disorders, and RLS-like symptoms (11, 12). However, the specific mechanisms must be further investigated. Other studies have focused on hypocretin-1 in the cerebrospinal fluid (CSF) because of its role in physiological rhythms. One study found that hypocretin-1 levels significantly increased during the evening in patients with early-onset RLS (13), while another one failed to find significant differences between hypocretin-1 levels of patients with RLS and control participants during the evening (14). Hypocretin-1 levels show a clear sinusoidal diurnal variation in healthy subjects (15), yet a study of patients with RLS failed to find evidence of a 24-h circadian rhythm in their hypocretin-1 levels (16). These conflicting results show that further research is needed. Genetic polymorphisms may also be a part of the pathogenic mechanism, with the *PTPRD, BTBD9*, and *MEIS1* genes as strong candidates of genetic risk factors for RLS (17, 18). Current pharmacological treatments for RLS include iron supplements, dopamine agonists, α2δ ligands, and opioids; however, these may be ineffective, and the dopamine agonists may even exacerbate the RLS symptoms in some refractory cases (19).

The evening/night-time worsening of symptoms greatly burdens patients with RLS by affecting their sleep quality, emotional state, and daytime function. Exploring the factors affecting RLS could help to clarify the pathophysiological mechanisms of this disorder and identify new therapeutic targets. Here, we illustrate some possible mechanisms that determine the circadian rhythm of RLS symptomatology, including iron deficiency, altered dopamine and hormone secretion, peripheral hypoxia, and impaired microvascular circulation function in the brain.

## 2. Circadian characteristics of restless legs syndrome

The circadian rhythm of RLS symptoms is clearly observed, since the unpleasant sensations and urge to move occur or become more severe in the evening/night than in the daytime. This rhythm always occurs in the early sleep stages, though if symptoms are severe, the night-time worsening may not be evident (1). The severity of sensory and motor RLS symptoms demonstrates a peak in the early stages of sleep (11 p.m.−4 a.m.) and a nadir during the initial waking period (9 a.m.−2 p.m.); additionally, though symptoms worsen as a result of sleep deprivation, the circadian rhythm appears to be independent of sleepiness, fatigue, or time since the last sleeping period (20–22).

The circadian rhythm of RLS symptoms is closely associated with the circadian clock mediated by the supraoptic nucleus of the hypothalamus. In a case of severe delayed sleep–wake phase disorder and simultaneous RLS in which sleep began at 8 a.m., symptoms of RLS still occurred according to the disrupted circadian rhythm (23). Other studies have reported that RLS symptoms still appeared at the usual time when shift workers first started their night shifts; as the workers' circadian rhythms adapted to the shift work schedule, the onset of symptoms was gradually delayed until it coincided with the time before sleep; moreover, westward cross-temporal flight results in a phase delay of RLS symptoms, whereas eastward cross-temporal flight instead causes an earlier onset of symptoms (24). This may be related to the effect of light on circadian rhythms, and it suggests that RLS symptom onset is independent of the duration of wakefulness; otherwise, cross-temporal flights would have the opposite effect. The circadian rhythm of RLS symptoms has also been significantly correlated with the core body temperature cycle; maximal symptoms occurred at the nadir of the core body-temperature rhythm (20–22). However, core body temperature rhythm does not appear to be altered in patients with RLS, which suggests that the basic circadian rhythm in patients of RLS is not disrupted or altered. Though it may be closely linked to the normal circadian rhythm, the circadian rhythm of RLS may not be directly controlled by the central circadian pacemaker, but instead associated with the diurnal variability of multiple biological factors, which are described in detail below.

## 3. The role of iron in RLS

### 3.1. Iron deficiency in the brain and circadian rhythm of iron

Iron is an essential trace element that plays an important role in energy metabolism, hormone synthesis, oxygen transfer, and immunity. It is a crucial component of hemoglobin and enzymes involved in electron transfer and neurotransmitter synthesis. Iron deficiency, especially brain iron, is thought to be the main factor in RLS pathophysiology. RLS prevalence is approximately nine times higher in patients with iron deficiency anemia than that in the general population (25), and intravenous iron supplementation therapy can improve RLS symptoms in some patients (26), which suggests a close association between iron deficiency and RLS symptoms. Patients with RLS have also demonstrated iron deficiencies in the brain; neuropathological autopsies (27) and ultrasound examinations (28) have found reduced iron levels in the substantia nigra of patients with RLS. Another study showed that CSF ferritin levels were significantly lower in patients with RLS than in controls (29). Additionally, MRI studies suggest that reduced iron in the brain may be more prevalent in these patients, particularly in the thalamus, substantia nigra, nucleus accumbens, and pallidum (30, 31). This cerebral iron deficiency may be due to altered expression of the iron regulator hepcidin and an iron management protein profile (such as transferrin and its receptor) in the epithelial cells of the choroid plexus and brain microvasculature of patients with RLS. Subsequent dysfunctions in iron transportation from the serum to the brain, cellular iron uptake, and blood iron homeostasis (32–35) result in brain iron deficiency. Due to complex regulatory mechanisms, measurements of peripheral iron status show no directly association with brain iron levels; systemic iron deficiency may lead to reduced brain iron only in specific regions and in certain individuals, and brain iron deficiency can also be present in individuals with normal peripheral iron (36), which create difficulties for assessing the degree of deficiency.

Circadian rhythm of iron is closely related to the circadian rhythm of RLS. Unger et al. reported a notable circadian rhythm of increasing iron levels in the ventral midbrain during sleep, with maximum levels in mid-morning and minimum levels during evening hours (37–39). Also, Earley et al. (29) found that CSF ferritin levels of patients with RLS at 10 a.m. (reflecting night-time brain iron status) were lower than those at 10 p.m. (reflecting afternoon brain iron status). Sleep deprivation may also decrease the mean level of iron and reduce the absolute and relative amplitudes of its circadian oscillations (40), supporting the link between RLS symptoms and sleep deprivation (21). Therefore, the nadir of iron levels due to circadian rhythms may be related to the occurrence or aggravation of RLS symptoms during the evening or at night.

### 3.2. Brain iron deficiency and dopaminergic system disorder

Iron deficiency also causes dysfunction of the dopaminergic system; dopaminergic disorders and the circadian rhythm of dopamine can then combine to cause a worsening of RLS symptoms at night. Patients with RLS demonstrate higher dopamine levels in the synaptic gap and lower levels in the intracellular space than those of controls; they also demonstrate reduced density of dopamine D2 receptors (D2R) in the putamen and dopamine reuptake receptors, according to previous studies using samples from CSF (41), post-mortem tissue (42), and animals (43). These dopaminergic changes result from iron deficiency in the brain, which can lead to high levels of dopamine through the hypoxia-inducible factor (HIF)-1 pathway (4) and downregulation of A1Rs (44). Similar dopaminergic changes were observed in iron-deficient mice and were reduced by administration of exogenous iron to the striatum (43, 45, 46). As with ferritin, dopamine expression also follows a circadian rhythm; levels in plasma typically peak at 8 a.m. and gradually decrease to 60% of the peak between 8 p.m. and 10 p.m., with the nadir occurring at ~3 a.m. (47).

Taken together, a model by Earley et al. showed the circadian rhythm of dopamine could cause the nocturnal symptom fluctuations that match the cycle of RLS symptom presence. Iron deficiency could contribute to a presynaptic hyperdopaminergic state, leading to the downregulation of D2Rs as postsynaptic feedback. Due to the diurnal variation of dopamine secretion, the feedback effects also vary dynamically. While this postsynaptic adaptation to increased dopamine stimulation is appropriate during the day when dopamine levels are higher, it appears to overcompensate in the evening when dopamine levels are lower. Excessive downregulation of postsynaptic D2Rs results in weak dopaminergic signaling when dopamine levels are low, creating a relative dopamine deficit despite an overall increase in dopamine. The intensity of the dopaminergic output signal thus varies with the circadian rhythm of dopamine levels, reaching its nadir in the late evening or after sleep onset. The weakening of dopaminergic signaling, particularly D2Rs, can reduce inhibition of painful stimuli and cause dysfunction in pain processing (48, 49) and may lead to increased release of glutamic acid (50, 51). Through these mechanisms, RLS symptoms may be triggered when the dopaminergic output signal falls below a critical threshold. This model could explain why patients experience the mildest RLS symptoms at late night or early morning and the most severe RLS symptoms at evening/night (4).

## 4. Peripheral hypoxia and impaired microvascular circulation

Since iron is crucial for oxygen transportation, hypoxia and impaired microcirculation are other potential mechanisms of RLS. In patients with RLS, HIF 1-α was higher in the substantia nigra, and HIF 2-α and vascular endothelial growth factor were increased in the microvessels, a possible sign of hypoxia due to iron deficiency (52, 53). Salminen et al. demonstrated that peripheral hypoxia was positively correlated with the presence and severity of RLS (54). Furthermore, impairment of microvascular circulation in patients with RLS has been demonstrated using bilateral great-toe laser Doppler flowmetry, whole-body thermography (55), and ultrasound imaging (56). Near-infrared light therapy is effective in ameliorating nocturnal symptoms by promoting nitric oxide release to improve vasodilation (57, 58), demonstrating the relationship between impaired microvascular circulation and RLS. A circadian rhythm of higher blood flow in the morning than in the evening was observed in the microcirculatory pattern of the tibialis anterior muscle in patients with primary RLS, but not in the control group (59). Thus, altered microcirculation might be another mechanism affecting the circadian rhythm in RLS.

## 5. Altered hormone secretion

### 5.1. Melatonin

Melatonin is an amine hormone produced by the pineal gland. Its secretion is influenced by light and has a well-defined circadian rhythm. Actively secreted in the darkness and suppressed during the day, it peaks in the middle of the night (2–4 a.m.) then gradually decreases. However, acute light exposure at night rapidly stops melatonin production (60), whereas in continuous darkness, the melatonin rhythm still remains. Michaud et al. demonstrated that melatonin secretion onset coincided with nocturnal symptom worsening in patients with RLS, and that salivary melatonin levels peaked ~2 h before the peak of sensory or motor symptom severity; moreover, melatonin promotes peripheral vasodilation in humans, leading to a decrease in core body temperature that is associated with the onset of RLS symptoms (20). However, Whittom et al. found that exogenous melatonin administration significantly worsened motor symptoms in patients with RLS, whereas inhibition of endogenous melatonin by strong light exposure at night improved sensory symptoms (61), which contrasted with melatonin's reported role in reducing neuropathic pain (62). Melatonin can inhibit the release of dopamine by suppressing the influx of calcium into stimulated nerve endings, while the suprachiasmatic nuclei modulates the sensitivity of dopaminergic neurons to melatonin (63); this may contribute to the relative evening/night-time dopamine deficits in RLS, exacerbating motor and sensory symptoms. Only a few studies have investigated this aspect; current results show that melatonin secretion patterns in patients with RLS are not significantly different than those in controls (20). Therefore, melatonin may be involved only as an upstream regulator of other pathways that influence the RLS circadian rhythms, though the specific mechanism requires further investigation.

### 5.2. Melanocortin

The alpha-melanocyte-stimulating hormone (α-MSH) and adrenocorticotropic hormone are centrally-acting melanocortins produced by hypothalamic pro-opiomelanocortin neurons. Their secretion also has a circadian rhythm, with a nocturnal peak occurring overnight (64). In menopausal women and rat models, exogenous melanocortin administration induced dose-dependent RLS-like symptoms, such as increased motor impulses, hyperactivity, sleep fragmentation, and periodic limb movements (65, 66). Melanocortins act as endogenous anti-opioids, and exogenous administration can lead to hyperalgesia in rats (67–69). Melanocortin secretion can be inhibited by dopamine, which blocks RNA synthesis of the melanocortin precursor pro-opiomelanocortin in the hypothalamus *via* D2Rs (70). The D2R downregulation seen in RLS may lead to hypersecretion of MSH (especially at night), potentially aggravating RLS symptoms. Therefore, disrupted MSH secretion is a likely mechanism underlying the night-time worsening of RLS symptoms; however, no study has directly examined the association between changes in endogenous MSH levels and the circadian rhythm of RLS symptoms. Further research is needed to determine the role of MSH in the pathophysiological mechanisms of RLS.

### 5.3. Thyroid-stimulating hormone

The thyroid-stimulating hormone (TSH) is a pituitary-derived hormone composed of α and β subunit genes. Regulated by the suprachiasmatic nuclei, TSH also follows a defined circadian rhythm of rising in the evening before sleep onset and peaking at night between 10 p.m. and 5 a.m., followed by a gradual decrease to a day-time minimum between 3 and 7 p.m. (71–73). This pattern is consistent with the circadian symptomatology in patients with RLS. Patients with hypothyroidism have poor sleep quality, as measured by prolonged sleep latency, long N1 and N2 phases, and short N3 and REM phases (74, 75).

TSH alterations may represent a pathophysiological mechanism of RLS. Geng et al. demonstrated that TSH levels are elevated in patients with RLS when compared to healthy controls and found TSH to be positively correlated with International Restless Legs Scale scores and negatively correlated with sleep quality (76). Ahmed et al. reported a correlation between RLS and hypothyroidism, with a significantly high prevalence of RLS in patients with hypothyroidism and a significantly high prevalence of hypothyroidism in patients with RLS (77).

The elevated TSH levels in patients with RLS may be related to the following mechanisms: first, dopamine can reduce TSH formation by inhibiting transcription and translation of the TSH β-subunit, mediated by the thyrotropin-releasing hormone (78); thus, the hypodopaminergic state in patients with RLS may lead to excessive TSH release. Second, iron deficiency can impair the heme-dependent thyroid peroxidase, reducing the synthesis of thyroid hormones and increasing TSH *via* feedback dysregulation (79, 80). The strong correlation between elevated TSH levels and RLS suggests that a TSH secretion imbalance may contribute to the circadian rhythmicity of RLS symptoms. However, further research is needed, as there are no studies specifically on the synchronization of RLS symptoms and the circadian rhythm of TSH secretion.

## 6. Treatment

Iron-replacement therapy is considered for all patients with RLS upon initial treatment and is administered if transferrin saturation levels are ≤ 45%. Oral iron treatment is generally safe and well-tolerated, and should be considered for serum ferritin levels of ≤ 75 μg/L in an adult or < 50 μg/L in a child; intravenous treatment is considered for adults with serum ferritin levels of 75–100 μg/L (36). Dopamine agonists and α2δ ligands are prescribed for use one to 2 h before the regular onset of RLS symptoms. Dopamine agonists bind to dopamine receptors and mimics the actions of dopamine to counteract the downregulation of dopaminergic output signal from the relative dopamine deficit during the evening, while α2δ ligands bind with high affinity to the α2δ-1 subunit of voltage-gated calcium channels, resulting in the reduced release of glutamic acid (81, 82). Meanwhile, dipyridamole is a novel and non-selective equilibrative nucleoside transporter 1/equilibrative nucleoside transporter 2 adenosine transporter antagonist that may counteract the hypoadenosinergic state in patients with RLS to reduce symptoms (83).

Non-pharmacologic treatment by pneumatic-compression devices or near-infrared light therapy can improve microvascular circulation during the night to relieve RLS symptoms (58, 84, 85). Standard acupuncture may be another safe alternative treatment (86). However, large-scale and adequately powered randomized controlled trials are required to estimate the efficacy of these approaches. Repetitive transcranial magnetic stimulation applied in the evening may provide relief for RLS symptoms by promoting dopamine release (87), while continuous transcutaneous direct current stimulation to the spinal cord may provide lasting symptom improvement (88). Yoga and night-time cold-water immersion of the legs can also reduce symptoms and severity, although the exact mechanism is unclear (89–91). Subclinical hypothyroidism may also require assessment before developing a treatment plan. Moreover, patients with RLS may have individualized circadian rhythms in which the exacerbation of symptoms occurs at fixed times or seasons; further study of these factors could guide clinical treatment (92).

## 7. Conclusion and prospect

**Figure 1:**
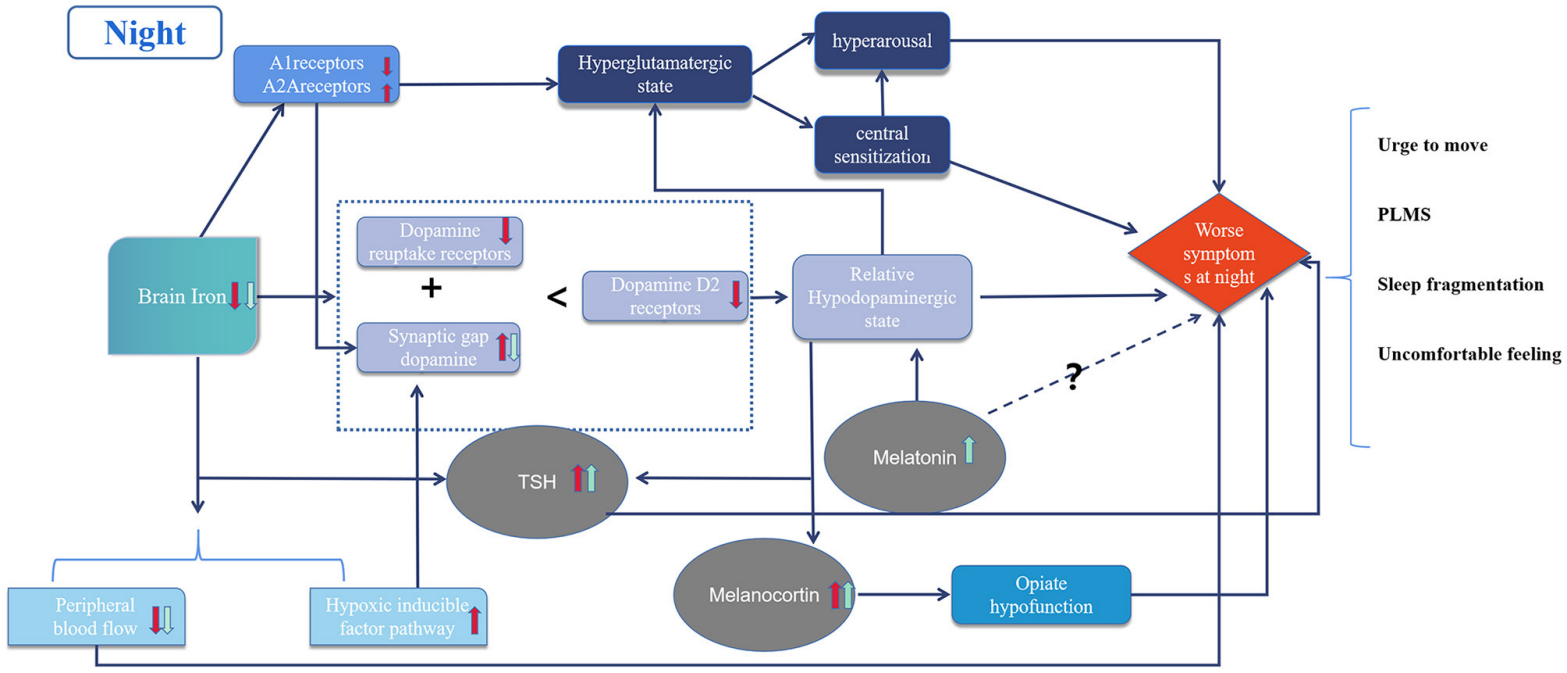
Diagram of circadian rhythm mechanism in restless legs syndrome. The red arrows represent pathological changes and the green arrows represent physiological changes in the night due to circadian rhythms. Dotted lines represent unknown mechanisms.

The circadian rhythm of RLS is related to several complex mechanisms (Figure 1). Initially, iron deficiency in the brain causes dopaminergic system dysfunction and hyperglutaminergic, hypoadenosinergic, and peripheral hypoxic states. These alterations result in downstream decreases of. dopamine-mediated inhibition, central nervous system sensitization, hyperarousal, and imbalance in the secretion of hormones with circadian rhythms, such as melatonin, melanocortin, and TSH. The pre-existing pathological imbalance is further aggravated by physiological oscillations from circadian rhythms, resulting in symptoms that worsen at night. Standard treatments include iron supplements, dopamine agonists, α2δ ligands and opioids; while some emerging therapies have demonstrated efficacy, further evidence of their mechanism of action is still needed.

In addition, other factors may affect the circadian rhythm in RLS. Recent research has also found evidence of a circadian rhythm in spinal cord excitability with RLS (93, 94), which may be related to the decrease in supraspinal inhibition mediated by the dopaminergic nucleus A11 of the hypothalamus (95). Meanwhile, the diurnal disturbances of the default mode network may also be related to the circadian rhythm of RLS, especially circadian connectivity changes to the thalamus (96). Although a hyperglutamatergic state is well-established in RLS patients, available studies were conducted in the morning (7) and no studies have attempted to clarify whether glutamate levels are further elevated at night owing to circadian oscillations of iron or dopamine. These factors may represent targets for further research to clarify the mechanisms of circadian rhythm in RLS. Accurate assessment of the circadian rhythm characteristics for each patient with RLS also requires further research.

## Author contributions

MT reviewed the literature, wrote drafts of the manuscript, and prepared the figure. QS adjusted the structure of the article and participated in the rewriting of some paragraphs during the revision of the manuscript. YZ and HL helped in designing and revising of the literature review. ZW supervised the work. YW and DW provided constructive advice. All authors contributed to the article and approved the submitted version.

## References

[1] Allen RP Picchietti DL Garcia-Borreguero D Ondo WG Walters AS Winkelman JW. Restless legs syndrome/Willis-Ekbom disease diagnostic criteria: updated International Restless Legs Syndrome Study Group (IRLSSG) consensus criteria–history, rationale, description, and significance. Sleep Med. (2014) 15:860–73. 10.1016/j.sleep.2014.03.025 25023924

[2] Gao X Ba DM Bagai K Liu G Ma C Walters AS. Treating restless legs syndrome was associated with low risk of cardiovascular disease: a cohort study with 3.4 years of follow-up. J Am Heart Assoc. (2021) 10:e018674. 10.1161/JAHA.120.018674 33550813 PMC7955352

[3] McDermott M Brown DL Chervin RD. Sleep disorders and the risk of stroke. Expert Rev Neurother. (2018) 18:523–31. 10.1080/14737175.2018.1489239 29902391 PMC6300163

[4] Earley CJ Connor J Garcia-Borreguero D Jenner P Winkelman J Zee PC. Altered brain iron homeostasis and dopaminergic function in restless legs syndrome (Willis–Ekbom disease). Sleep Med. (2014) 15:1288–301. 10.1016/j.sleep.2014.05.009 25201131

[5] Cho YW Kang M-S Kim KT Do SY Lim J-G Lee SY. Quantitative sensory test for primary restless legs syndrome/Willis-Ekbom disease using the current perception threshold test. Sleep Med. (2017) 30:19–23. 10.1016/j.sleep.2016.03.003 28215248

[6] Goulart LI Delgado Rodrigues RN Prieto Peres MF. Restless legs syndrome and pain disorders: what's in common? Curr Pain Headache Rep. (2014) 18:461. 10.1007/s11916-014-0461-0 25249423

[7] Allen RP Barker PB Horská A Earley CJ. Thalamic glutamate/glutamine in restless legs syndrome: increased and related to disturbed sleep. Neurology. (2013) 80:2028–34. 10.1212/WNL.0b013e318294b3f6 23624560 PMC3716406

[8] Ferré S Quiroz C Guitart X Rea W Seyedian A Moreno E. Pivotal role of adenosine neurotransmission in restless legs syndrome. Front Neurosci. (2017) 11:722. 10.3389/fnins.2017.00722 29358902 PMC5766678

[9] Rodrigues MS Ferreira SG Quiroz C Earley CJ García-Borreguero D Cunha RA. Brain iron deficiency changes the stoichiometry of adenosine receptor subtypes in cortico-striatal terminals: implications for restless legs syndrome. Molecules. (2022) 27:1489. 10.3390/molecules27051489 35268590 PMC8911604

[10] Walters AS Ondo WG Zhu W Le W. Does the endogenous opiate system play a role in the Restless Legs Syndrome? A pilot post-mortem study. J Neurol Sci. (2009) 279:62–5. 10.1016/j.jns.2008.12.022 19167016

[11] Lyu S DeAndrade MP Mueller S Oksche A Walters AS Li Y. Hyperactivity, dopaminergic abnormalities, iron deficiency and anemia in an in vivo opioid receptors knockout mouse: implications for the restless legs syndrome. Behav Brain Res. (2019) 374:112123. 10.1016/j.bbr.2019.112123 31376441 PMC6728912

[12] Lyu S DeAndrade MP Unger EL Mueller S Oksche A Walters AS. Mu opioid receptor knockout mouse: phenotypes with implications on restless legs syndrome. J Neurosci Res. (2020) 98:1532–48. 10.1002/jnr.24637 32424971 PMC7430552

[13] Allen RP Mignot E Ripley B Nishino S Earley CJ. Increased CSF hypocretin-1 (orexin-A) in restless legs syndrome. Neurology. (2002) 59:639–41. 10.1212/WNL.59.4.639 12196669

[14] Stiasny-Kolster K Mignot E Ling L Möller JC Cassel W Oertel WH. CSF hypocretin-1 levels in restless legs syndrome. Neurology. (2003) 61:1426–9. 10.1212/01.WNL.0000094196.50155.38 14638970

[15] Salomon RM Ripley B Kennedy JS Johnson B Schmidt D Zeitzer JM. Diurnal variation of cerebrospinal fluid hypocretin-1 (Orexin-A) levels in control and depressed subjects. Biol Psychiatry. (2003) 54:96–104. 10.1016/S0006-3223(02)01740-7 12873798

[16] Poceta JS Parsons L Engelland S Kripke DF. Circadian rhythm of CSF monoamines and hypocretin-1 in restless legs syndrome and Parkinson's disease. Sleep Med. (2009) 10:129–33. 10.1016/j.sleep.2007.11.002 18207455

[17] Lyu S Doroodchi A Xing H Sheng Y DeAndrade MP Yang Y. BTBD9 and dopaminergic dysfunction in the pathogenesis of restless legs syndrome. Brain Struct Funct. (2020) 225:1743–60. 10.1007/s00429-020-02090-x 32468214 PMC7429108

[18] Jiménez-Jiménez FJ Alonso-Navarro H García-Martín E Agúndez JAG. Genetics of restless legs syndrome: an update. Sleep Med Rev. (2018) 39:108–21. 10.1016/j.smrv.2017.08.002 29033051

[19] Silber MH Buchfuhrer MJ Earley CJ Koo BB Manconi M Winkelman JW. The management of restless legs syndrome: an updated algorithm. Mayo Clin Proc. (2021) 96:1921–37. 10.1016/j.mayocp.2020.12.026 34218864

[20] Michaud M Dumont M Selmaoui B Paquet J Fantini ML Montplaisir J. Circadian rhythm of restless legs syndrome: relationship with biological markers. Ann Neurol. (2004) 55:372–80. 10.1002/ana.10843 14991815

[21] Hening WA Walters AS Wagner M Rosen R Chen V Kim S. Circadian rhythm of motor restlessness and sensory symptoms in the idiopathic restless legs syndrome. Sleep. (1999) 22:901–12. 10.1093/sleep/22.7.901 10566908

[22] Trenkwalder C Hening WA Walters AS Campbell SS Rahman K Chokroverty S. Circadian rhythm of periodic limb movements and sensory symptoms of restless legs syndrome. Mov Disord. (1999) 14:102–10. 10.1002/1531-8257(199901)14:1<102::AID-MDS1017>3.0.CO;2-E 9918351

[23] Ulfberg J Sielaff B Grote L. A case of severe delayed sleep–wake phase disorder and simultaneous restless legs syndrome. Sleep Vigilance. (2019) 3:157–8. 10.1007/s41782-019-00071-7

[24] Garcia-Borreguero D Larrosa O de la Llave Y. Circadian aspects in the pathophysiology of the restless legs syndrome. Sleep Med. (2002) 3:S17–21. 10.1016/S1389-9457(02)00143-0 14592162

[25] Allen RP Auerbach S Bahrain H Auerbach M Earley CJ. The prevalence and impact of restless legs syndrome on patients with iron deficiency anemia. Am J Hematol. (2013) 88:261–4. 10.1002/ajh.23397 23494945

[26] Earley CJ Horská A Mohamed MA Barker PB Beard JL Allen RP. A randomized, double-blind, placebo-controlled trial of intravenous iron sucrose in restless legs syndrome. Sleep Med. (2009) 10:206–11. 10.1016/j.sleep.2007.12.006 18280205 PMC2703581

[27] Connor JR Boyer PJ Menzies SL Dellinger B Allen RP Ondo WG. Neuropathological examination suggests impaired brain iron acquisition in restless legs syndrome. Neurology. (2003) 61:304–9. 10.1212/01.WNL.0000078887.16593.12 12913188

[28] Godau J Schweitzer KJ Liepelt I Gerloff C Berg D. Substantia nigra hypoechogenicity: definition and findings in restless legs syndrome. Mov Disord. (2007) 22:187–92. 10.1002/mds.21230 17133515

[29] Earley CJ Connor JR Beard JL Clardy SL Allen RP. Ferritin levels in the cerebrospinal fluid and restless legs syndrome: effects of different clinical phenotypes. Sleep. (2005) 28:1069–75. 10.1093/sleep/28.9.1069 16268375

[30] Rizzo G Manners D Testa C Tonon C Vetrugno R Marconi S. Low brain iron content in idiopathic restless legs syndrome patients detected by phase imaging. Mov Disord. (2013) 28:1886–90. 10.1002/mds.25576 23780623

[31] Godau J Klose U Di Santo A Schweitzer K Berg D. Multiregional brain iron deficiency in restless legs syndrome. Mov Disord. (2008) 23:1184–7. 10.1002/mds.22070 18442125

[32] Chenini S Delaby C Rassu A-L Barateau L Vialaret J Hirtz C. Hepcidin and ferritin levels in restless legs syndrome: a case-control study. Sci Rep. (2020) 10:11914. 10.1038/s41598-020-68851-0 32681031 PMC7367854

[33] Mizuno S Mihara T Miyaoka T Inagaki T Horiguchi J. CSF iron, ferritin and transferrin levels in restless legs syndrome. J Sleep Res. (2005) 14:43–7. 10.1111/j.1365-2869.2004.00403.x 15743333

[34] Connor JR Wang XS Patton SM Menzies SL Troncoso JC Earley CJ. Decreased transferrin receptor expression by neuromelanin cells in restless legs syndrome. Neurology. (2004) 62:1563–7. 10.1212/01.WNL.0000123251.60485.AC 15136682

[35] Connor JR Ponnuru P Wang X-S Patton SM Allen RP Earley CJ. Profile of altered brain iron acquisition in restless legs syndrome. Brain. (2011) 134:959–68. 10.1093/brain/awr012 21398376 PMC3069701

[36] Allen RP Picchietti DL Auerbach M Cho YW Connor JR Earley CJ. Evidence-based and consensus clinical practice guidelines for the iron treatment of restless legs syndrome/Willis-Ekbom disease in adults and children: an IRLSSG task force report. Sleep Med. (2018) 41:27–44. 10.1016/j.sleep.2017.11.1126 29425576

[37] Casale G Migliavacca A Bonora C Zurita IE de Nicola P. Circadian rhythm of plasma iron, total iron binding capacity and serum ferritin in arteriosclerotic aged patients. Age Ageing. (1981) 10:115–8. 10.1093/ageing/10.2.115 7246335

[38] Third JLHC Ryan MD Sothern RB Dawson S McCormick JB Hoffman HS. Circadian distribution of iron and ferritin in serum of healthy and type 2 diabetic males. Clin Ter. (2006) 157:35–40. Available online at: https://pubmed.ncbi.nlm.nih.gov/16669550/16669550

[39] Unger EL Earley CJ Thomsen LL Jones BC Allen RP. Effects of IV iron isomaltoside-1000 treatment on regional brain iron status in an iron-deficient animal. Neuroscience. (2013) 246:179–85. 10.1016/j.neuroscience.2013.04.049 23660192

[40] Kuhn E Brodan V. Changes in the circadian rhythm of serum iron induced by a 5-day sleep deprivation. Eur J Appl Physiol Occup Physiol. (1982) 49:215–22. 10.1007/BF02334070 6889498

[41] Allen RP Connor JR Hyland K Earley CJ. Abnormally increased CSF 3-Ortho-methyldopa (3-OMD) in untreated restless legs syndrome (RLS) patients indicates more severe disease and possibly abnormally increased dopamine synthesis. Sleep Med. (2009) 10:123–8. 10.1016/j.sleep.2007.11.012 18226951 PMC2655320

[42] Connor JR Wang X-S Allen RP Beard JL Wiesinger JA Felt BT. Altered dopaminergic profile in the putamen and substantia nigra in restless leg syndrome. Brain. (2009) 132:2403–12. 10.1093/brain/awp125 19467991 PMC2732265

[43] Earley CJ Jones BC Ferré S. Brain-iron deficiency models of restless legs syndrome. Exp Neurol. (2022) 356:114158. 10.1016/j.expneurol.2022.114158 35779614 PMC9357217

[44] Ferré S Bonaventura J Zhu W Hatcher-Solis C Taura J Quiroz C. Essential control of the function of the striatopallidal neuron by pre-coupled complexes of adenosine A(2A)-dopamine D receptor heterotetramers and adenylyl cyclase. Front Pharmacol. (2018) 9:243. 10.3389/fphar.2018.00243 29686613 PMC5900444

[45] Unger EL Bianco LE Jones BC Allen RP Earley CJ. Low brain iron effects and reversibility on striatal dopamine dynamics. Exp Neurol. (2014) 261:462–8. 10.1016/j.expneurol.2014.06.023 24999026 PMC4318655

[46] Bianco LE Unger EL Earley CJ Beard JL. Iron deficiency alters the day-night variation in monoamine levels in mice. Chronobiol Int. (2009) 26:447–63. 10.1080/07420520902820905 19360489

[47] Sowers JR Vlachakis N. Circadian variation in plasma dopamine levels in man. J Endocrinol Invest. (1984) 7:341–5. 10.1007/BF03351014 6501806

[48] Bravo L Llorca-Torralba M Berrocoso E Micó JA. Monoamines as drug targets in chronic pain: focusing on neuropathic pain. Front Neurosci. (2019) 13:1268. 10.3389/fnins.2019.01268 31942167 PMC6951279

[49] Sheng HY Qu CL Huo FQ Du JQ Tang JS. D2-like but not D1-like dopamine receptors are involved in the ventrolateral orbital cortex-induced antinociception: a GABAergic modulation mechanism. Exp Neurol. (2009) 215:128–34. 10.1016/j.expneurol.2008.09.018 18952080

[50] González S Rangel-Barajas C Peper M Lorenzo R Moreno E Ciruela F. Dopamine D4 receptor, but not the ADHD-associated D4.7 variant, forms functional heteromers with the dopamine D2S receptor in the brain. Mol Psychiatry. (2012) 17:650–62. 10.1038/mp.2011.93 21844870 PMC3219836

[51] Yepes G Guitart X Rea W Newman AH Allen RP Earley CJ. Targeting hypersensitive corticostriatal terminals in restless legs syndrome. Ann Neurol. (2017) 82:951–60. 10.1002/ana.25104 29171915 PMC5739944

[52] Patton SM Ponnuru P Snyder AM Podskalny GD Connor JR. Hypoxia-inducible factor pathway activation in restless legs syndrome patients. Eur J Neurol. (2011) 18:1329–35. 10.1111/j.1468-1331.2011.03397.x 21985026

[53] Wåhlin-Larsson B Ulfberg J Aulin KP Kadi F. The expression of vascular endothelial growth factor in skeletal muscle of patients with sleep disorders. Muscle Nerve. (2009) 40:556–61. 10.1002/mus.21357 19623635

[54] Salminen AV Rimpilä V Polo O. Peripheral hypoxia in restless legs syndrome (Willis-Ekbom disease). Neurology. (2014) 82:1856–61. 10.1212/WNL.0000000000000454 24789861

[55] Anderson KN Di Maria C Allen J. Novel assessment of microvascular changes in idiopathic restless legs syndrome (Willis-Ekbom disease). J Sleep Res. (2013) 22:315–21. 10.1111/jsr.12025 23397977

[56] Koh SY Kim MS Lee SM Hong JM Yoon JH. Impaired vascular endothelial function in patients with restless legs syndrome: a new aspect of the vascular pathophysiology. J Neurol Sci. (2015) 359:207–10. 10.1016/j.jns.2015.10.041 26671114

[57] Mitchell UH. Use of near-infrared light to reduce symptoms associated with restless legs syndrome in a woman: a case report. J Med Case Rep. (2010) 4:286. 10.1186/1752-1947-4-286 20731851 PMC2936319

[58] Mohammadi MM Raygani AAV Ghobadi A Samadzadeh S Salari N. Effect of near-infrared light therapy based on acupoints on the severity of restless legs syndrome in patients undergoing hemodialysis: a single-blind, randomized controlled trial. Clin Med Res. (2018) 16:1–8. 10.3121/cmr.2018.1389 29776916 PMC6108511

[59] Oskarsson E Wåhlin-Larsson B Ulfberg J. Reduced daytime intramuscular blood flow in patients with restless legs syndrome/Willis-Ekbom disease. Psychiatry Clin Neurosci. (2014) 68:640–3. 10.1111/pcn.12170 24521165

[60] Brzezinski A. Melatonin in humans. N Engl J Med. (1997) 336:186–95. 10.1056/NEJM199701163360306 8988899

[61] Whittom S Dumont M Petit D Desautels A Adam B Lavigne G. Effects of melatonin and bright light administration on motor and sensory symptoms of RLS. Sleep Med. (2010) 11:351–5. 10.1016/j.sleep.2009.12.008 20226733

[62] Kuthati Y Lin S-H Chen I-J Wong C-S. Melatonin and their analogs as a potential use in the management of neuropathic pain. J Formos Med Assoc. (2019) 118:1177–86. 10.1016/j.jfma.2018.09.017 30316678

[63] Zisapel N. Melatonin-dopamine interactions: from basic neurochemistry to a clinical setting. Cell Mol Neurobiol. (2001) 21:605–16. 10.1023/A:1015187601628 12043836 PMC11533843

[64] Kim Y Carpenter AM Gregg KJ Shahnaz Z Carr JA. Diurnal variation in alpha-melanocyte-stimulating hormone content of various brain regions and plasma of the Texas toad, Bufo speciosus. Gen Comp Endocrinol. (1995) 98:50–6. 10.1006/gcen.1995.1043 7781964

[65] Kastin AJ Kullander S Borglin NE Dahlberg B Dyster-Aas K Krakau CE. Extrapigmentary effects of melanocyte-stimulating hormone in amenorrhoeic women. Lancet. (1968) 1:1007–10. 10.1016/S0140-6736(68)91113-6 4171803

[66] Koo BB Feng P Dostal J Strohl KP. Alpha-melanocyte stimulating hormone and adrenocorticotropic hormone: an alternative approach when thinking about restless legs syndrome? Mov Disord. (2008) 23:1234–42. 10.1002/mds.22035 18464280 PMC7350876

[67] Gispen WH Van Wimersma Greidanus TB Waters-Ezrin C Zimmermann E Krivoy WA De Wied D. Influence of peptides on reduced response of rats to electric footshock after acute administration of morphine. Eur J Pharmacol. (1975) 33:99–105. 10.1016/0014-2999(75)90143-0 170120

[68] Sandman CA Kastin AJ. Intraventricular administration of MSH induces hyperalgesia in rats. Peptides. (1981) 2:231–3. 10.1016/S0196-9781(81)80040-X 7291044

[69] Alvaro JD Tatro JB Duman RS. Melanocortins and opiate addiction. Life Sci. (1997) 61:1–9. 10.1016/S0024-3205(97)00029-5 9200663

[70] Zhou Y Spangler R Yuferov VP Schlussmann SD Ho A Kreek MJ. Effects of selective D1- or D2-like dopamine receptor antagonists with acute “binge” pattern cocaine on corticotropin-releasing hormone and proopiomelanocortin mRNA levels in the hypothalamus. Brain Res Mol Brain Res. (2004) 130:61–7. 10.1016/j.molbrainres.2004.07.008 15519677

[71] Brabant G Prank K Ranft U Schuermeyer T Wagner TO Hauser H. Physiological regulation of circadian and pulsatile thyrotropin secretion in normal man and woman. J Clin Endocrinol Metab. (1990) 70:403–9. 10.1210/jcem-70-2-403 2105332

[72] Kalsbeek A Fliers E Franke AN Wortel J Buijs RM. Functional connections between the suprachiasmatic nucleus and the thyroid gland as revealed by lesioning and viral tracing techniques in the rat. Endocrinology. (2000) 141:3832–41. 10.1210/endo.141.10.7709 11014240

[73] Weeke J Gundersen HJ. Circadian and 30 minutes variations in serum TSH and thyroid hormones in normal subjects. Acta Endocrinol. (1978) 89:659–72. 10.1530/acta.0.0890659 716774

[74] Shekhar S Hall JE Klubo-Gwiezdzinska J. The hypothalamic pituitary thyroid axis and sleep. Curr Opin Endocr Metab Res. (2021) 17:8–14. 10.1016/j.coemr.2020.10.002 34322645 PMC8315115

[75] Song L Lei J Jiang K Lei Y Tang Y Zhu J. The association between subclinical hypothyroidism and sleep quality: a population-based study. Risk Manag Healthc Policy. (2019) 12:369–74. 10.2147/RMHP.S234552 31908553 PMC6927586

[76] Geng C Yang Z Kong X Xu P Zhang H. Association between thyroid function and disease severity in restless legs syndrome. Front Neurol. (2022) 13:974229. 10.3389/fneur.2022.974229 36034269 PMC9412235

[77] Ahmed N Kandil M Elfil M Jamal A Koo BB. Hypothyroidism in restless legs syndrome. J Sleep Res. (2021) 30:e13091. 10.1111/jsr.13091 32483857

[78] Shupnik MA Greenspan SL Ridgway EC. Transcriptional regulation of thyrotropin subunit genes by thyrotropin-releasing hormone and dopamine in pituitary cell culture. J Biol Chem. (1986) 261:12675–9. 10.1016/S0021-9258(18)67144-3 2427524

[79] Kawicka A Regulska-Ilow B Regulska-Ilow B. Metabolic disorders and nutritional status in autoimmune thyroid diseases. Postepy Hig Med Dosw. (2015) 69:80–90. 10.5604/17322693.1136383 25614676

[80] Zimmermann MB. The influence of iron status on iodine utilization and thyroid function. Annu Rev Nutr. (2006) 26:367–89. 10.1146/annurev.nutr.26.061505.111236 16602928

[81] Winkelman JW Armstrong MJ Allen RP Chaudhuri KR Ondo W Trenkwalder C. Practice guideline summary: treatment of restless legs syndrome in adults: report of the guideline development, dissemination, and implementation Subcommittee of the American Academy of Neurology. Neurology. (2016) 87:2585–93. 10.1212/WNL.0000000000003388 27856776 PMC5206998

[82] Faulkner MA. Use of α2δ ligands for restless legs syndrome/Willis Ekbom Disease. CNS Drugs. (2018) 32:149–59. 10.1007/s40263-018-0502-z 29480463

[83] Garcia-Borreguero D Guitart X Garcia Malo C Cano-Pumarega I Granizo JJ Ferré S. Treatment of restless legs syndrome/Willis-Ekbom disease with the non-selective ENT1/ENT2 inhibitor dipyridamole: testing the adenosine hypothesis. Sleep Med. (2018) 45:94–7. 10.1016/j.sleep.2018.02.002 29680437

[84] Lettieri CJ Eliasson AH. Pneumatic compression devices are an effective therapy for restless legs syndrome: a prospective, randomized, double-blinded, sham-controlled trial. Chest. (2009) 135:74–80. 10.1378/chest.08-1665 19017878

[85] Mitchell UH Myrer JW Johnson AW Hilton SC. Restless legs syndrome and near-infrared light: an alternative treatment option. Physiother Theory Pract. (2011) 27:345–51. 10.3109/09593985.2010.511440 20977377

[86] Harrison EG Keating JL Morgan PE. Non-pharmacological interventions for restless legs syndrome: a systematic review of randomised controlled trials. Disabil Rehabil. (2019) 41:2006–14. 10.1080/09638288.2018.1453875 29561180

[87] Nardone R Sebastianelli L Versace V Brigo F Golaszewski S Pucks-Faes E. Contribution of transcranial magnetic stimulation in restless legs syndrome: pathophysiological insights and therapeutical approaches. Sleep Med. (2020) 71:124–34. 10.1016/j.sleep.2019.12.009 32088150

[88] Wang L Liu C Hou Y Zhan S Zhang Z Wang J. Altered cortical gray matter volume and functional connectivity after transcutaneous spinal cord direct current stimulation in idiopathic restless legs syndrome. Sleep Med. (2020) 74:254–61. 10.1016/j.sleep.2020.07.026 32862009

[89] Innes KE Selfe TK Montgomery C Hollingshead N Huysmans Z Srinivasan R. Effects of a 12-week yoga versus a 12-week educational film intervention on symptoms of restless legs syndrome and related outcomes: an exploratory randomized controlled trial. J Clin Sleep Med. (2020) 16:107–19. 10.5664/jcsm.8134 31957638 PMC7053002

[90] Innes KE Selfe TK Agarwal P Williams K Flack KL. Efficacy of an eight-week yoga intervention on symptoms of restless legs syndrome (RLS): a pilot study. J Altern Complement Med. (2013) 19:527–35. 10.1089/acm.2012.0330 23270319 PMC3673587

[91] Jafarimanesh H Vakilian K Mobasseri S. Thermo-therapy and cryotherapy to decrease the symptoms of restless leg syndrome during the pregnancy: a randomized clinical trial. Complement Ther Med. (2020) 50:102409. 10.1016/j.ctim.2020.102409 32444058

[92] Ingram DG Plante DT. Seasonal trends in restless legs symptomatology: evidence from Internet search query data. Sleep Med. (2013) 14:1364–8. 10.1016/j.sleep.2013.06.016 24152798

[93] Dafkin C Green A Olivier B McKinon W Kerr S. Plantar reflex excitability is increased in the evening in restless legs syndrome patients. Neurosci Lett. (2017) 660:74–8. 10.1016/j.neulet.2017.09.027 28917979

[94] Dafkin C Green A Olivier B McKinon W Kerr S. Circadian variation of flexor withdrawal and crossed extensor reflexes in patients with restless legs syndrome. J Sleep Res. (2018) 27:e12645. 10.1111/jsr.12645 29164719

[95] Lanza G Bachmann CG Ghorayeb I Wang Y Ferri R Paulus W. Central and peripheral nervous system excitability in restless legs syndrome. Sleep Med. (2017) 31:49–60. 10.1016/j.sleep.2016.05.010 27745789

[96] Ku J Lee YS Chang HW Earley CJ Allen RP Cho YW. Diurnal variation of default mode network in patients with restless legs syndrome. Sleep Med. (2018) 41:1–8. 10.1016/j.sleep.2017.09.031 29425573

